# Students as teachers: the value of peer-led teaching

**DOI:** 10.1007/s40037-013-0094-8

**Published:** 2013-11-28

**Authors:** Rele Ologunde, Rasheed Rabiu

**Affiliations:** Imperial College School of Medicine, Imperial College London, Exhibition Road, London, SW7 2AZ UK

## Students as teachers: the value of peer-led teaching

During the time spent at medical school, students may be presented with various opportunities to engage in teaching and medical education. Often these opportunities are passed up or indeed overlooked all together. However, the value of developing teaching skills and engaging in such activities for the dissemination, application and translation of medical knowledge is of great importance to the progress of all trainees. The CanMEDS physician competency framework highlights the role of the scholar as being one of the six constituents integral to the development of the medical expert [1]. This role of the scholar encompasses the expectation that students and trainees will develop proficiency in their teaching skills along with other important clinical, developmental and training specific criteria.

The General Medical Council recognizes that it is a developmental imperative to mentor the core skills of teaching in the doctors of tomorrow [2]. Beyond the scope of continued professional development, teaching as a student provides an opportunity to begin to contribute to the future medical workforce, many of whom may be future colleagues. In addition, with the increase in patient involvement in decision-making, future doctors must appreciate that good teaching strategies and communication skills will play an important role in doctor-patient interactions.

Peer-assisted learning (PAL) is defined as ‘People of similar social groupings who are not professional teachers helping each other to learn and learning themselves by teaching’ [3]. This definition clearly depicts PAL as an experience that both the tutor and tutee can learn from. The benefits of PAL are not surprising. Both the teacher and students share a similar knowledge base and learning experience; the dynamic interplay between these and other factors facilitates fluid communication between both groups whilst also providing an environment for learning and teaching that is optimal. Furthermore, it could be argued that student-tutors are better at judging the learner’s knowledge and consequently better at anticipating the learning difficulties that may arise during teaching.

Participation in peer-led teaching can be a valuable part of undergraduate and postgraduate medical education for both the learners and the teachers. The peer-led environment supports a favourable learning experience and builds effective teaching skills that will be useful in future careers as physicians.
